# Supplementary material for: A new approach to cultural scripts of trauma sequelae assessment: The sample case of Switzerland
Source: PLoS One. 2024 Apr 16;19(4):e0301645. doi: 10.1371/journal.pone.0301645 (PMC11020718; doi:10.1371/journal.pone.0301645)
Supplement: S1 Table — (DOCX) [file pone.0301645.s002.docx]

# S1 Table

*MAXQDA-derived code structure, number of coded segments, and example participant statements*

| **Main category** | **Subcodes** | **Coded segments** | **Example participant statements** |
| --- | --- | --- | --- |
| Cognitions | Self-deprecation | 100 | «... I started thinking a lot about why this happened to me. The logical step in my head was, yeah, because I'm so bad. I deserved it/ it doesn't happen to others, so there must be something extremely bad about me that it happened to me.» (survivor) |
|  | Insecure self-concept | 14 | «Yes, you never really know what's going to happen next. Also with feelings, with your own feelings. Or also how you would react to an / some event.» (survivor) |
|  | Urge to function and perform | 33 | «The right to exist of those patients that I have in mind is that they define themselves very much by their performance, by functioning and performing as part of society, that seems to be central as a justification for some of them.» (expert) |
|  | Urge to control | 19 | «When I realize that I start having feelings, I think ‘oh no, it cannot happen. Like / like if I would now allow these feelings, something extremely bad would happen.» (survivor) |
|  | Avoid speaking about own suffering | 13 | «This reminds me in this context / what was very strong for both / is this vow of silence. It accompanied and shaped them very strongly and when one of them for the first time reported about the abuse in therapy, she immediately decompensated.» (expert) |
|  | Trivializing own emotions | 11 | «They also trivialize traumatic events to some extent or, because they always say, 'Yes, others have had that too, so I don't have to react in such a way that it massively limits me.» (expert) |
|  | Endure pain and suffering | 10 | «So that, yes, something [painful] is there, but you still carry on and don't show it much to the outside world.» (expert) |
| Affects | Emotion regulation difficulties | 36 | «I'll say a hundred times beforehand, 'I don't want to burst. I am going to burst right now.' And at some point, I burst and then I'm very mean and then I have to call my psychologist the next day because I have such a guilty conscience.» (survivor) |
|  | Shame | 13 | «There is also a lot of shame, towards relatives or society in general.» (expert) |
|  | Guilt | 10 | I certainly give others a lot more space than I do (double). In various areas. And if I take the space, I immediately feel guilty. (survivor) |
|  | Fear | 7 | Hm, there are also moments that are associated with fear and the feeling of being at the mercy of others. So, hm helplessness, that you somehow can't defend yourself. Being at the mercy of others and also the fear that something bad could happen now. (survivor) |
|  | Anger | 4 | And then it's mostly directed / so it's like a switch. I'd actually be angry at someone else, but in the end, I direct this anger against myself. (survivor) |
|  | *Other emotions* | 28 | Then I sometimes think, if you only knew how weak I am. But I would never show it to the outside world. (...) (survivor)  Yes I just get the feeling, I have I have like failed in a way (twice). I didn't fight back. I have found no way (..) to get out of it. (survivor) |
| Worldviews | World is dangerous | 8 | … what I think happens with most people is simply that the world somehow becomes more dangerous, right? So, it's somehow / hm, just somehow the feeling of threat. One is no longer self-confident, one expects / yes behind every corner something is expected to befall you. (expert) |
|  | Negative view of humanity | 19 | Actually, I find humans to be the worst. I know there are good ones and I've had the chance many times, but, for example, I don't trust anyone 100%. Not even me, not even my father. (survivor) |
| Interpersonal | Mistrust | 16 | So actually, I don't trust anyone. And building trust is very difficult. There are maybe one or two people I trust and the rest seem to trust me, I don't really know why (laughs). I always have to make sure that I create a bit of distance because it quickly becomes too close for me. (survivor) |
|  | Difficulties to establish relationships | 16 | Just out of self-protection, they just don't go into it so much / don’t get involved with other people. (expert) |
|  | Fear of rejection | 9 | So, the x few years I've avoided it completely. I've never let it happen in any way. Hm, it is also connected with / actually with fear (...) to be rejected, actually. (survivor) |
|  | Toxic relationships | 8 | Both of the ones I have in mind have entered into very harmful relationships over the years / for a very long time / relationships that were not as massively abusive as the original relationships in the family, but still, yes, very violent, also psychologically. (expert) |
|  | Difficulties to stand up for oneself | 8 | Or if I notice that someone I don't know that well needs help, for example moving apartments, and [this person] asks me, I have the feeling, 'oh I can't say no now', although I actually don't want to help at all. (survivor) |
|  | Never ask for help | 6 | Exactly, or asking for help is also difficult. It doesn't matter if your arm is hurt and you're not feeling well (smiles). I'd rather try for two hours to see if I can tie my shoes one-handed before I ask someone to tie them for me. But I managed to do it (laughs). (survivor) |
|  | Social withdrawal | 4 | … a withdrawal as well, or. You don't take part in family reunions and so on anymore, because maybe there you have already experienced an outbreak or something and then you avoid it (expert) |
| Embitterment | No wish to participate in life | 4 | …and the other is the thought (...) 'actually, today is a good day to (..) die'. And that every day (survivor) |
|  | Lack of meaning | 2 | I think trauma tears such a big hole in a life, hm (...) that for a while you have no possibility at all to concentrate on meaningfulness / what is good for you, what are you looking for, where would you like to go / because there is something else, bigger, which (6) / yes, in that moment it is about survival and, I think, even when the trauma itself, when the traumatic experience or the experiences are over, somehow it is still about survival for quite a long time. And I think, as long as you fight for survival, (..) it's like not important / you cannot think about meaningfulness / or look for meaning. (survivor) |
| Body-related | Psychosomatic pain | 15 | So, (...) I have (..) / erm just rarely, if there are extreme situations or if somehow panic arises or something, I have pain in certain parts of the body, which like now erm / are medically inexplicable or make no sense. So it's really triggered psychologically. (survivor) |
|  | Self-harming behavior | 12 | Yes, self-injury (inc.). (7) And sports. Extreme sports. (survivor) |
|  | Other psychosomatic symptoms | 11 | Have pain and other physical symptoms that conventional medicine does not classify» (expert) |
|  | Lack of energy, tiredness, exhaustion | 11 | And I find things like that extremely difficult. And incredibly exhausting, until you have built it up again to some extent and realize again 'Okay, now, slowly I can breathe again and I can get my bearings' (inc.) and so on. And that is now, for example, something that requires an extremely large amount of energy. (survivor) |
|  | Rejection of own body | 10 | …but that there are almost always object relationships to the body. So the body is sort of the favorite enemy or the main opponent or something. (expert) |
|  | Disconnected from one’s body | 8 | I believe that after taking up climbing, for the first time in my life I really felt my body. (survivor) |
|  | Eating problems | 6 | I've often noticed that I've often had episodes in which I just don't eat. (survivor) |
| Growth | Developed specific strengths | 23 | … like (...) a certain strength, strengths that I have developed through the experience. That like / even if things maybe don't go so well, that I can still say, hey, I've made it this far, I've already gotten through so much, to put it plainly, I'll get through it now. (survivor) |
|  | Gratefulness and appreciation | 12 | And the other thing is that I understand a lot of people who are in difficult situations. (4) And to whom I really am able to offer support. (survivor) |
|  | Experiencing more intensely | 9 | But just this / or just, I don't know, when it rained outside, the smell and the mood and I can draw from that for three days and that's just something where yeah / where I think I wouldn't otherwise / yeah. (survivor) |
