# Supplementary material for: A new approach to cultural scripts of trauma sequelae assessment: The sample case of Switzerland
Source: PLoS One. 2024 Apr 16;19(4):e0301645. doi: 10.1371/journal.pone.0301645 (PMC11020718; doi:10.1371/journal.pone.0301645)
Supplement: S2 Table — (DOCX) [file pone.0301645.s003.docx]

# S2 Table

*Swiss Cultural Scripts of Trauma Inventory*

| **Instructions**: The following questionnaire lists different reactions and changes that people may experience after a traumatic event. Please read each description carefully and indicate how strongly it applies to your experience since the traumatic event.  **Response** **format**: 5-point Likert scale: 0 = not at all, 1 = a little, 2 = moderately, 3 = strongly, 4 = very strongly. | | | |
| --- | --- | --- | --- |
| No | Item | PRE-CSTI | New |
| **Changes in cognitions and affects** | | | |
| 1 | I feel like I don’t know myself anymore. (Not knowing myself) | x |  |
| 2 | I think I will never be able to feel normal emotions again. (No normal emotions) | x |  |
| 3 | I think that I am a weak person. (Weakness) | x |  |
| 4 | I feel ashamed. (Shame) | x |  |
| 5 | I have feelings of guilt. (Guilt) | x |  |
| 6 | I feel disgust. (Disgust) | x |  |
| 7 | I think I should have prevented the trauma. (Prevent trauma) | x |  |
| 8 | I think I am generally damaged or bad. (Being damaged) | x |  |
| 9 | I feel like a failure. (Failure) |  | x |
| 10 | I feel deep grief about what has happened to me. (Grief) |  | x |
| 11 | I feel helpless or powerless in certain situations. (Helplessness) |  | x |
| 12 | My anger is most likely directed at myself. (Anger against myself) |  | x |
| 13 | I try to prove my worth by performing particularly well. (Urge to perform) |  | x |
| 14 | I believe that I have to work and function at all costs. (Urge to function) |  | x |
| 15 | It is important for me to appear normal to the outside world. (Appear normal) |  | x |
| 16 | It is important for me to have control over my feelings/body. (Control over myself) |  | x |
| 17 | I think that my pain/suffering is nothing special. (Suffering is normal) |  | x |
| 18 | I have difficulties in perceiving and/or realizing my own needs. (Perceive needs) |  | x |
| 19 | I try to avoid thoughts, feelings, and situations related to the trauma. (Avoidance) |  | x |
| 20 | I often feel anxious. (Anxiety) |  | x |
| 21 | I am overwhelmed by my feelings. (Overwhelm) |  | x |
| 22 | I am thinking about taking my own life. (Suicidality) |  | x |
| 23 | There are situations in which I no longer have access to my feelings. (No access to feelings) |  | x |
| **Changed world views** | |  |  |
| 24 | I think that nothing good can happen to me anymore. (Pessimism) | x |  |
| 25 | I think the world is a dangerous place. (Dangerous world) | x |  |
| 26 | I have to be especially careful because you never know what can happen next. (Caution) | x |  |
| 27 | I feel like I do not have a place in the world. (Not having a place) | x |  |
| 28 | I find it difficult to adapt to external changes. (Adjustment difficulties) | x |  |
| 29 | I think that other people can’t be trusted. (Mistrust) | x |  |
| **Interpersonal changes** | |  |  |
| 30 | My relationships have been damaged or challenged. (Damaged relations) | x |  |
| 31 | I am a burden to others. (Being a burden) | x |  |
| 32 | I do not share my thoughts with others, not even with friends. (No shared feelings) | x |  |
| 33 | I am most comfortable alone. (Prefer being alone) |  | x |
| 34 | I don't feel lovable. (Not lovable) |  | x |
| 35 | When others see me as I really am, they reject me. (Fear of rejection) |  | x |
| 36 | I find it difficult to ask others for help. (Not getting help) |  | x |
| 37 | I tend to enter into relationships that are not good for me. (Dysfunctional relationships) |  | x |
| **Motivational changes** | |  |  |
| 38 | I am troubled by fears that I may never achieve my dreams. (Failed dreams) | x |  |
| 39 | My life has no meaning. (Meaninglessness) | x |  |
| 40 | I lack the energy for life. (Lack of energy) |  | x |
| **Growth** | |  |  |
| 41 | I discovered that I am stronger than I thought I was. (More strength) | x |  |
| 42 | I notice that I understand other people in need better. (More understanding) | x |  |
| 43 | I have (further) developed personal skills. (Skills developed) |  | x |
| 44 | I experience nature particularly intensively. (Nature) |  | x |
| **Body-related changes** | |  |  |
| 45 | I experience an overwhelming feeling of exhaustion and physical weakness. (Exhaustion) | x |  |
| 46 | I have difficulties with physical intimacy. (Difficulties with intimacy) | x |  |
| 47 | I intentionally hurt myself to cope with distressing thoughts and feelings. (Self-harm) | x |  |
| 48 | I resort to alcohol or other substances to deal with distressing thoughts and feelings. (Substance use) | x |  |
| 49 | I suffer from back pain much more frequently. (Back pain) | x |  |
| 50 | I suffer from stomach aches much more frequently. (Stomachache) | x |  |
| 51 | I suffer from headaches much more frequently. (Headache) | x |  |
| 52 | I get dizzy more often. (Dizziness) | x |  |
| 53 | There are situations in which I no longer feel my body. (No body feeling) | x |  |
| 54 | I do not feel well in my body. (Body discomfort) |  | x |
| 55 | I have pain that is probably psychological. (Psychotropic pain) |  | x |
| 56 | I have sleep disturbances. (Sleep disturbance) |  | x |
| 57 | I often have problems with eating. (Eating problems) |  | x |
