# Supplementary material for: A new approach to cultural scripts of trauma sequelae assessment: The sample case of Switzerland
Source: PLoS One. 2024 Apr 16;19(4):e0301645. doi: 10.1371/journal.pone.0301645 (PMC11020718; doi:10.1371/journal.pone.0301645)
Supplement: S3 Table — (DOCX) [file pone.0301645.s004.docx]

S3 Table

*PRE-CSTI items not included in Swiss CSTI*

| **Changes in cognitions and affects** |
| --- |
| I feel dead inside. |
| My beliefs / my faith have / has been challenged by the event. |
| My values have changed. |
| I think too much, which I suffer from. |
| **Changed world views** |
| I feel let down by the good forces in the world. |
| **Interpersonal changes** |
| The balance in my family has been disrupted. |
| I think that you can never know who will harm you. |
| I feel isolated and set apart from others. |
| I am incapable of fulfilling my duties towards my family and others. |
| I believe that the envy of others also plays a role in my current situation. |
| After what happened, I am worried about offending others through my behaviour (e.g., blushing) or appearance  (e.g., looks, body odour). |
| I have difficulties saving face with others. |
| **Embitterment** |
| I think that no one should have to live this way. |
| It all seems so unfair. |
| I think that nothing will ever make up for all that I have gone through. |
| I feel as if I have been robbed of something very precious. |
| I can’t believe this has happened to me. |
| I get angry about something that wasn’t very important. |
| I have thoughts or fantasies about hurting someone. |
| I wish that I wasn’t so angry all the time. |
| I have no clear sense of direction in life. |
| I have difficulties forgiving people who have hurt me. |
| I think if another person hurts you, it’s alright to get back at him or her. |
| When somebody offends me, sooner or later I retaliate. |
| **Growth** |
| People have become a priority. |
| I have changed my priorities about what is important in life. |
| I have a greater appreciation for the value of my own life. |
| I have stronger religious or spiritual faith. |
| I think that my mental disorder is caused by malfunctions in my vital organs. |
| **Body-related changes** |
| I experience fits of uncontrollable crying or screaming. |
| I experience a feeling of heat in the chest rising to the head. |
| I experience attacks of dizziness, palpitations and shortness of breath. |
| I suffer from a disruption of the energy flow in my body. |
