# Supplementary material for: A new approach to cultural scripts of trauma sequelae assessment: The sample case of Switzerland
Source: PLoS One. 2024 Apr 16;19(4):e0301645. doi: 10.1371/journal.pone.0301645 (PMC11020718; doi:10.1371/journal.pone.0301645)
Supplement: S4 Table — (DOCX) [file pone.0301645.s005.docx]

# S4 Table

*Compilation of the temporal-causal connections of script elements*

| Serial number | **Script** | **Origin** |
| --- | --- | --- |
| 1 | Guilt 🡪 self-deprecation | Expert |
| 2 | Guilt 🡨🡪 anger 🡨🡪 sadness/grief | Survivor |
| 3 | Overwhelmed by intense emotions 🡪 dissociation | Survivor |
| 4 | Shame 🡪 self-deprecation 🡪 caution/reluctance in interpersonal context | Survivor |
| 5 | Self-deprecation 🡪 self-harm/suicidality | Expert |
| 6 | Sense of threat 🡪 caution/reluctance in interpersonal context | Expert |
| 7 | Guilt 🡪 shame | Survivor |
| 8 | Guilt 🡪 shame 🡪 self-deprecation 🡪 helplessness | Expert |
| 9 | Self-depreciation 🡪 guilt | Survivor |
| 10 | Strong emotions 🡪 dissociation 🡪 helplessness | Survivor |
| 11 | Urge to function 🡪 shame (when not functioning) 🡪 maintain façade of normalcy 🡪 hide suffering | expert |
| 12 | Anger 🡪 direct anger inwards 🡪 self-deprecation | survivor |
| 13 | Urge to function 🡪 maintain façade of normalcy 🡪 Hide/endure suffering 🡪 self-deprecation | expert |
| 14 | Anger 🡪 direct anger inwards OR at other 🡪 shame | survivor |
| 15 | Trivialize own suffering 🡪 self-deprecation | expert |
| 16 | Anger 🡪 overwhelmed by intense emotions 🡪 control/suppress emotions | Survivor |
| 17 | Guilt/shame 🡪 urge to function | expert |
| 18 | Mistrust 🡪 caution/reluctance (interpersonal) | survivor |
| 19 | Overwhelmed by intense emotions 🡪 panic | survivor |
| 20 | Not feeling one’s body 🡪 self-harm | survivor |
| 21 | Mistrust 🡪 urge to control relationships 🡪 caution/reluctance (interpersonal) | survivor |
| 22 | Urge to function 🡪 hide/endure suffering | expert |
| 23 | Anger 🡪 overwhelmed by intense emotions 🡨🡪 control/suppress emotions 🡪 self-deprecation | survivor |
| 24 | Urge to function 🡪 trivialize own suffering 🡪 social withdrawal (when no longer functioning) | expert |
| 25 | Anger OR sadness 🡪 overwhelmed by intense emotions 🡨🡪 control/suppress | survivor |
| 26 | Urge to function 🡪 endure own suffering | expert |
| 27 | Urge to control situation/life/world 🡪 urge to function | expert |
| 28 | Urge to control situation/life/world 🡪 urge to function 🡪 endure own suffering 🡪 psychosomatic symptoms | expert |
| 29 | Self-deprecation 🡪 negative body image | survivor |
| 30 | Toxic relationships 🡪 mistrust | survivor |
| 31 | Dissociation 🡪 not feeling one’s body 🡪 negative body image (not trust body) | survivor |
| 32 | Overwhelmed by intense emotions 🡪 dissociation 🡪 not feeling one’s body | survivor |
| 33 | Urge to control emotions/urge to control situation/urge to control relationships 🡪 psychosomatic symptoms | expert |
| 34 | Panic 🡪 psychosomatic symptoms | survivor |
| 35 | Urge to control situation/own life/world 🡪 trivialize own suffering 🡪 psychosomatic symptoms | expert |
| 36 | Sense of threat 🡪 psychosomatic symptoms | survivor |
| 37 | Overwhelmed by intense emotions 🡪 tiredness/exhaustion | survivor |
| 38 | Mistrust 🡪 unable to protect own needs 🡪 urge to control situation/relationships 🡪 social withdrawal | survivor |
| 39 | Urge to function 🡪 unable to perceive own needs 🡪 tiredness exhaustion | expert |
| 40 | Overwhelmed by intense emotions 🡪 self-deprecation | patient |
| 41 | Caution/reluctance (interpersonal) 🡪 guilt 🡪 sense of threat 🡪 Caution/reluctance (interpersonal) | patient |
| 42 | Mistrust 🡪 urge to control situation/relationships | patient |
| 43 | Self-deprecation 🡪 trivialize own suffering 🡪 urge to function | patient |
| 44 | self-deprecation 🡪 caution/reluctance (interpersonal and situational) | patient |
| 45 | Anger 🡪 direct anger inwards 🡪 psychosomatic symptoms OR social withdrawal OR substance abuse | patient |
| 46 | Self-deprecation 🡪 social withdrawal | patient |
| 47 | Urge to function 🡪 self-deprecation 🡪 fear of rejection | patient |
| 48 | Self-deprecation 🡪 guilt 🡪 unable to protect own needs | patient |
| 49 | Self-deprecation 🡪 guilt 🡪 unable to protect own needs (different example for previous script) | patient |
| 50 | Self-deprecation 🡪 fear of rejection | patient |
| 51 | Control, suppress emotions 🡪 substance abuse | expert |
| 52 | Self-deprecation 🡪 fear of rejection 🡪 social withdrawal | patient |
| 53 | Self-deprecation 🡪 unable to protect own needs |  |
| 54 | Unable to protect own needs 🡪 sadness/grief 🡪 anger 🡪 direct anger inwards 🡪 self-deprecation | patient |
| 55 | Tiredness, exhaustion 🡪self-deprecation | patient |
| 56 | Negative body image 🡪self-deprecation | patient |
| 57 | Self-deprecation 🡪 fear of rejection 🡪 urge to function/(over)compensate | patient |
| 58 | Self-deprecation 🡨🡪 urge to function/overcompensate 🡪 helplessness | patient |
| 59 | Hide/endure suffering 🡪 social withdrawal 🡪 toxic relationships | expert |
| 60 | Overwhelmed by strong emotions 🡪 control/suppress emotions 🡪 helplessness 🡪 tiredness | patient |
| 61 | Urge to function 🡪 control/suppress emotions 🡪 overwhelmed by emotions | expert |
| 62 | Self-deprecation 🡪 urge to function/overcompensate 🡪 tiredness/exhaustion | survivor |
| 63 | Caution/reluctance (interpersonal) 🡪 Urge to control situation/relationships 🡪 panic 🡪 overwhelmed by intense emotions 🡪 psychosomatic symptoms | survivor |
| 64 | Trivialize own suffering 🡪 overwhelmed by intense emotions | survivor |
| 65 | Urge to control situation 🡪 eating problems 🡪 negative body image | expert |
| 66 | Overwhelmed by strong emotions 🡪 self-deprecation 🡪 eating problems 🡪 tiredness/exhaustion | survivor |
| 67 | Overwhelmed by strong emotions 🡪 tiredness/exhaustion 🡪 helplessness | survivor |
| 68 | Self-deprecation 🡪 urge to function | survivor |
| 69 | Maintain façade of normalcy 🡪 Hide/endure suffering 🡪 caution/reluctance (interpersonal) | expert |
| 70 | Guilt and shame 🡪 self-deprecation | expert |
