# Supplementary material for: A new approach to cultural scripts of trauma sequelae assessment: The sample case of Switzerland
Source: PLoS One. 2024 Apr 16;19(4):e0301645. doi: 10.1371/journal.pone.0301645 (PMC11020718; doi:10.1371/journal.pone.0301645)
Supplement: S5 Table — (DOCX) [file pone.0301645.s006.docx]

# S5 Table

*CSTI network “nodes” and corresponding CSTI Items*

| **Nodes in the network** | **Nr.** | **CSTI Item** |
| --- | --- | --- |
| Self-deprecation | 3 | I think that I am a weak person. (Weakness) |
|  | 8 | I think I am generally damaged or bad. (Being damaged) |
|  | 9 | I feel like a failure. (Failure) |
|  | 27 | I feel like I do not have a place in the world. (Not having a place) |
|  | 31 | I am a burden to others. (Being a burden) |
|  | 34 | I don't feel lovable. (Not lovable) |
|  | 38 | I am troubled by fears that I may never achieve my dreams. (Failed dreams) |
|  | 6 | I feel disgust. (Disgust) 🡪 when related to oneself. |
|  | 39 | My life has no meaning. (Meaninglessness) 🡪 feeling unworthy. |
| Shame | 4 | I feel ashamed. (Shame) |
| Guilt | 5 | I have feelings of guilt. (Guilt) |
|  | 7 | I think I should have prevented the trauma. (Prevent trauma) |
|  |  |  |
| Urge to function/  (over)compensate | 13 | I try to prove my worth by performing particularly well. (Urge to perform) |
|  |  |  |
|  | 14 | I believe that I have to work and function at all costs (Urge to function) |
| Maintain façade of normalcy | 15 | It is important for me to appear normal to the outside world. (Appear normal) |
| Hide and endure suffering | 18 | I have difficulties in perceiving and/or realizing my own needs. (Perceive needs) |
|  | 36 | I find it difficult to ask others for help. (Not getting help) |
| Trivialize own suffering | 17 | I think that my pain/suffering is nothing special. (Suffering is normal) |
|  |  |  |
| Overwhelmed by intense emotions; Panic | 21 | I am overwhelmed by my feelings. (Overwhelm) |
|  | 2 | I think I will never be able to feel normal emotions again. (No normal emotions) |
| Control/suppress emotions | 16 | It is important for me to have control over my feelings/body. (Control over myself) |
|  | 19 | I try to avoid thoughts, feelings, and situations related to the trauma. (Avoidance) |
| Dissociation | 23 | There are situations in which I no longer have access to my feelings. (No access to feelings) |
|  | 53 | There are situations in which I no longer feel my body. (No body feeling) |
| Tiredness/Exhaustion | 40 | I lack the energy for life. (Lack of energy) |
|  | 45 | I experience an overwhelming feeling of exhaustion and physical weakness. (Exhaustion) |
|  | 28 | I find it difficult to adapt to external changes. (Adjustment difficulties) |
|  | 24 | I think that nothing good can happen to me anymore. (Pessimism) |
|  | 22 | I am thinking about taking my own life. (Suicidality) |
|  | 39 | My life has no meaning. (Meaninglessness) |
| Anger; Direct anger inwards | 12 | My anger is most likely directed at myself. (Anger against myself) |
| Sadness/grief | 10 | I feel deep grief about what has happened to me. (Grief) |
| Helplessness | 11 | I feel helpless or powerless in certain situations. (Helplessness) |
|  | 1 | I feel like I don’t know myself anymore. (Not knowing myself) |
| Sense of threat | 20 | I often feel anxious. (Anxiety) |
|  | 25 | I think the world is a dangerous place. (Dangerous world) |
|  | 26 | I have to be especially careful because you never know what can happen next. (Caution) |
|  | 29 | I think that other people can’t be trusted. (Mistrust) |
| Caution/reluctance in interpersonal context | 32 | I do not share my thoughts with others, not even with friends. (No shared feelings) |
|  | 33 | I am most comfortable alone. (Prefer being alone) |
|  | 30 | My relationships have been damaged or challenged. (Damaged relations) |
|  | 37 | I tend to enter into relationships that are not good for me. (Dysfunctional relationships) |
| Fear of rejection | 35 | When others see me as I really am, they reject me. (Fear of rejection) |
| Urge to control social situation/relationships | 36 | I find it difficult to ask others for help. (Not getting help) |
